# Modular mimicry and engagement of the Hippo pathway by Marburg virus VP40: Implications for filovirus biology and budding

**DOI:** 10.1371/journal.ppat.1008231

**Published:** 2020-01-06

**Authors:** Ziying Han, Shantoshini Dash, Cari A. Sagum, Gordon Ruthel, Chaitanya K. Jaladanki, Corbett T. Berry, Michael P. Schwoerer, Nina M. Harty, Bruce D. Freedman, Mark T. Bedford, Hao Fan, Sachdev S. Sidhu, Marius Sudol, Olena Shtanko, Ronald N. Harty

**Affiliations:** 1 Department of Pathobiology, School of Veterinary Medicine, University of Pennsylvania, Philadelphia, Pennsylvania, United States of America; 2 Department of Epigenetics & Molecular Carcinogenesis, M.D. Anderson Cancer Center, University of Texas, Smithville, Texas, United States of America; 3 Department of Physiology and Mechanobiology Institute at National University of Singapore, Institute for Molecular and Cell Biology, IMCB, and Bioinformatics Institute, Agency for Science, Technology and Research (A*STAR), Singapore; 4 Department of Molecular Genetics, University of Toronto, Toronto, Ontario, Canada; 5 Texas Biomedical Research Institute, San Antonio, Texas, United States of America; National Institute of Allergy and Infectious Diseases, UNITED STATES

## Abstract

Ebola (EBOV) and Marburg (MARV) are members of the *Filoviridae* family, which continue to emerge and cause sporadic outbreaks of hemorrhagic fever with high mortality rates. Filoviruses utilize their VP40 matrix protein to drive virion assembly and budding, in part, by recruitment of specific WW-domain-bearing host proteins *via* its conserved PPxY Late (L) domain motif. Here, we screened an array of 115 mammalian, bacterially expressed and purified WW-domains using a PPxY-containing peptide from MARV VP40 (mVP40) to identify novel host interactors. Using this unbiased approach, we identified Yes Associated Protein (YAP) and Transcriptional co-Activator with PDZ-binding motif (TAZ) as novel mVP40 PPxY interactors. YAP and TAZ function as downstream transcriptional effectors of the Hippo signaling pathway that regulates cell proliferation, migration and apoptosis. We demonstrate that ectopic expression of YAP or TAZ along with mVP40 leads to significant inhibition of budding of mVP40 VLPs in a WW-domain/PPxY dependent manner. Moreover, YAP colocalized with mVP40 in the cytoplasm, and inhibition of mVP40 VLP budding was more pronounced when YAP was localized predominantly in the cytoplasm rather than in the nucleus. A key regulator of YAP nuclear/cytoplasmic localization and function is angiomotin (Amot); a multi-PPxY containing protein that strongly interacts with YAP WW-domains. Interestingly, we found that expression of PPxY-containing Amot rescued mVP40 VLP egress from either YAP- or TAZ-mediated inhibition in a PPxY-dependent manner. Importantly, using a stable Amot-knockdown cell line, we found that expression of Amot was critical for efficient egress of mVP40 VLPs as well as egress and spread of authentic MARV in infected cell cultures. In sum, we identified novel negative (YAP/TAZ) and positive (Amot) regulators of MARV VP40-mediated egress, that likely function in part, via competition between host and viral PPxY motifs binding to modular host WW-domains. These findings not only impact our mechanistic understanding of virus budding and spread, but also may impact the development of new antiviral strategies.

## Author summary

By screening an array of 115 mammalian WW-domains with the PPxY motif from MARV VP40 (mVP40), we identified YAP1 and TAZ, transcriptional effectors of the Hippo pathway, as mVP40 interactors, and demonstrated that ectopically expressed YAP1 or TAZ inhibited budding of mVP40 virus-like particles (VLPs) in a WW-domain/PPxY dependent manner. Angiomotin (Amot), a multi-PPxY containing regulator of YAP1 nuclear/cytoplasmic localization and function, rescued mVP40 VLP egress from either YAP1- or TAZ-mediated inhibition in a PPxY-dependent manner. Indeed, endogenous Amot expression was critical for egress of mVP40 VLPs and authentic MARV. In sum, we have revealed a link between the Hippo pathway and filovirus egress by identifying negative (YAP/TAZ) and positive (Amot) regulators of MARV VP40-mediated egress.

## Introduction

Filoviruses (Ebola [EBOV] and Marburg [MARV]) are high-priority, emerging pathogens, for which there are no approved vaccines nor therapeutic agents. As EBOV and MARV have been reported to cross epithelial and endothelial cell barriers and re-emerge months later in immunologically privileged sites including the CNS, semen, and retina, a better understanding of viral-host interactions that contribute to the transmission and pathogenesis of these deadly viruses is more critical than ever [1–4]. VP40 is the major structural protein that uniquely and independently directs assembly and egress of both virus-like particles (VLPs) and infectious filovirus virions. To accomplish this, VP40 uses highly conserved Late (L) budding domains (L-domains) that function to recruit or hijack a select set of host proteins that facilitate late stages of virus-cell separation [5–13]. For example, the well-described PPxY L-domain motif mediates the recruitment of a series of host HECT-family E3 ubiquitin ligases *via* one or more of their cognate WW-domains to facilitate egress [9,10,14–20]. In general, viral PPxY/WW-domain interactions involving host E3 ligases are believed to promote mono-ubiquitinylation of the viral matrix proteins [11–13,21–39], which allows the viral matrix protein to engage the ESCRT machinery to facilitate virus-cell separation at the plasma membrane [5,7,11–13,21,23–27,31,35–45].

There is a built-in degree of specificity of PPxY/WW-domain binding such that specific PPxY containing proteins will only interact physically and functionally with select WW-domain partners [46–49]. To date, much of the focus on viral L-domain motifs has been on their recruitment of host E3 ubiquitin ligases and/or the ESCRT pathway to advance virus egress and spread. To identify other cellular pathways employing WW-domain bearing proteins that could potentially affect filovirus egress and spread, and to further identify the overall complement of WW domains and its host proteins capable of binding to filovirus VP40 PPxY motifs, we probed a GST-fused array of 115 mammalian WW domains that were bacterially expressed, purified and fixed on solid support [50] with WT or mutant PPxY containing peptides from mVP40. Results from our screen identified two novel mVP40 PPxY interactors; YAP (Yes Associated Protein) and TAZ (Transcriptional co-Activator with PDZ-binding motif, also known as WWTR1 [WW domain-containing Transcription Regulator 1]).

YAP/TAZ are functional paralogues and the final downstream transcriptional effectors of the Hippo signaling pathway that regulates organ development, cell proliferation, migration, survival, and homeostasis [51–57]. Intriguingly, the Hippo signaling pathway is regulated by a series of PPxY/WW-domain modular interactions which lead ultimately to either sequestration or degradation of phosphorylated YAP in the cytoplasm (Hippo on), or to localization of unphosphorylated YAP in the nucleus where it upregulates a series of TEAD genes involved in cell growth and proliferation (Hippo off) [58]. One of the key regulators of YAP/TAZ localization and Hippo pathway activity is angiomotin (Amot), a member of the angiomotin family (Amot, AmotL1, and AmotL2), all of which contain multiple PPxY motifs at their N-termini [59–65]. Indeed, the PPxY/WW-domain interaction between Amot and YAP is well-characterized and reported to affect multiple processes in the cell [60–62,64–69].

In this report, we sought to determine whether the PPxY/WW-domain interaction between mVP40 and YAP/TAZ affected mVP40-mediated budding, and whether the PPxY motifs of Amot could compete with mVP40 for YAP1 binding to diminish these effects. Indeed, we found that: 1) co-expression of YAP1 or TAZ with mVP40 inhibited egress of mVP40 VLPs, 2) mVP40 VLP egress was not inhibited as significantly by YAP1 localized predominantly in the nucleus, 3) Amot expression positively regulated mVP40 VLP budding and rescued budding of mVP40 VLPs in the presence of YAP1 in a PPxY-dependent manner, and 4) Amot expression enhanced release and spread of authentic MARV in cell culture. These findings provide evidence that both PPxY and WW-domain proteins of the Hippo signaling pathway act as novel interactors and/or regulators of MARV VP40-mediated egress and spread. Moreover, these results highlight the potential domain mimicry and competitive interplay between host and viral proteins that can result in either a favorable or unfavorable outcome for the virus. The consequences of this intersection between virus budding and Hippo signaling not only may impact virus egress and spread, but also influence the biology and pathogenesis of filoviruses.

## Results

### Identification of YAP and TAZ as WW-domain interactors with the PPxY motif of mVP40

**Fig 1:**
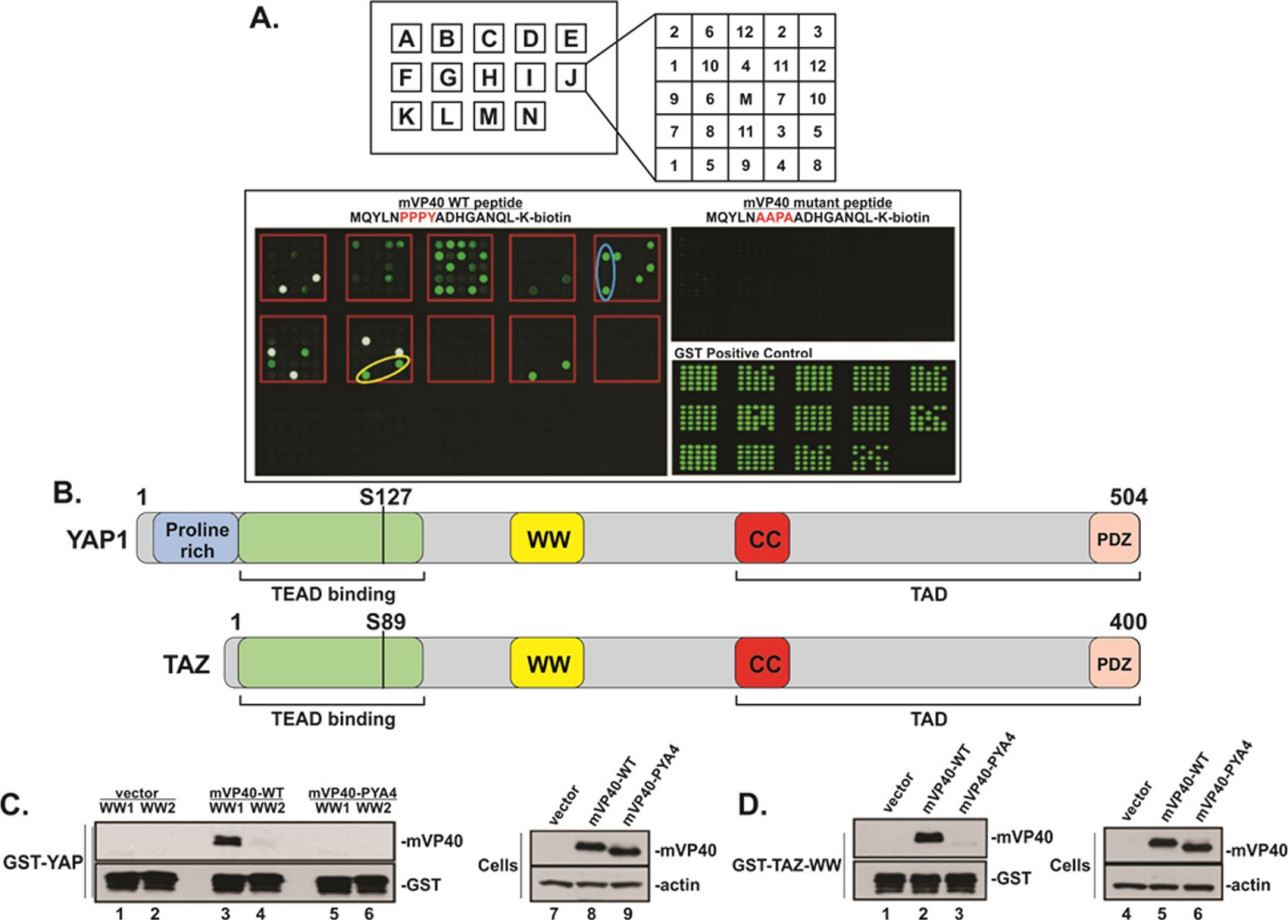
Screening array of modular proteins and GST-pulldown assays. **A)** Twelve different GST-SH3 and GST-WW domain fusion proteins per lettered box were arrayed in duplicate as indicated in enlarged box J (Top). The center sample (M) in each box represents GST alone as a negative control. The array was screened with biotinylated PPxY-WT or PPxY-mutant peptides of MARV VP40. Representative data for mVP40-WT peptide (MQYLNPPPYADHGANQL-[Lys-Biotin]) (50μg), and mVP40-mutant peptide (MQYLNAAPAADHGANQL-[Lys-Biotin]) (50μg) are shown. The mVP40-mutant peptide did not interact with any GST-SH3 or GST-WW domain fusion protein (center), whereas the mVP40-WT peptide interacted with specific GST-WW domain fusion proteins, including YAP1-1-WW (blue oval) and TAZ-WW (yellow oval). A positive control for expression of all GST-WW fusion proteins is shown. **B)** Schematic diagrams of human YAP1 and TAZ with key domains highlighted are shown. Numbers represent amino acids. Serine 127 and serine 89 are key sites for phosphorylation. TEAD = TEA DNA binding domain; WW = WW domain, CC = coiled coil domain; TAD = transcriptional activation domain; PDZ = PSD-95/Dlg1/ZO-1 domain. **C)** Input levels of purified GST-YAP-WW1 and GST-YAP-WW2 fusion proteins were shown to be consistent for all samples as determined by Western blotting using anti-GST antiserum (lanes 1–6). Only mVP40-WT was pulled down by GST-YAP-WW1 (lane 3). Expression levels for mVP40-WT, mVP40-PYA4, and actin in transfected HEK293T cells are shown (lanes 7–8). **D)** Input levels of purified GST-TAZ-WW fusion protein were shown to be consistent for all samples as determined by Western blotting using anti-GST antiserum (lanes 1–3). Only mVP40-WT was pulled down by GST-TAZ-WW (lane 2). Expression levels for mVP40-WT, mVP40-PYA4, and actin in transfected HEK293T cells are shown (lanes 4–6).

We used fluorescently-labeled, biotinylated peptides containing WT or mutated PPxY motifs from mVP40 to screen an array composed of 115 WW-domains and 40 SH3-domains that are derived from human proteins to detect novel host interactors (Fig 1A). We identified a select number of host WW-domain interactors (see Supplementary S1 Table) using the WT mVP40 peptide, whereas the mVP40 PPxY mutant peptide did not interact with any WW- or SH3-domain on the array (Fig 1A). Among the interactors identified were several HECT family E3 ubiquitin ligases that we and others have identified previously, as well as two novel WW-domain interactors; YAP1 and TAZ (Fig 1A and 1B).

There are two major isoforms of YAP; YAP1-1 contains one WW-domain (WW1), whereas YAP1-2 contains two WW-domains (WW1 and WW2) [70]. To validate further the interaction between the mVP40 PPxY motif and the WW-domains of YAP and TAZ, we used GST-YAP-WW1, GST-YAP-WW2, and GST-TAZ-WW fusion proteins to pulldown full-length mVP40-WT or mVP40-PYA4 mutant proteins expressed in HEK293T cells (Fig 1C and 1D). We found that mVP40-WT interacted strongly with WW1 of YAP1 (Fig 1C, lane 3), and weakly with WW2 of YAP1-2 (Fig 1C, lane 4). mVP40-PYA4 did not interact with either YAP WW1 or WW2 domains (Fig 1C, lanes 5 + 6). Similarly, we found that mVP40-WT, but not mVP40-PYA4, interacted with GST-TAZ-WW (Fig 1D, lanes 2 + 3). Consistent levels of expression for all GST fusion proteins (Fig 1C, lanes 1–6, and Fig 1D, lanes 1–3; anti-GST), mVP40-WT (Fig 1C, lane 8 and Fig 1D, lane 5), and mVP40-PYA4 (Fig 1C, lane 9 and Fig 1D, lane 6) were confirmed by Western blotting. Taken together, these data indicate that the PPxY motif of mVP40 can interact specifically and robustly with YAP-WW1 and TAZ-WW domains.

### Ectopic Expression of YAP1 or TAZ inhibits mVP40 VLP budding

**Fig 2:**
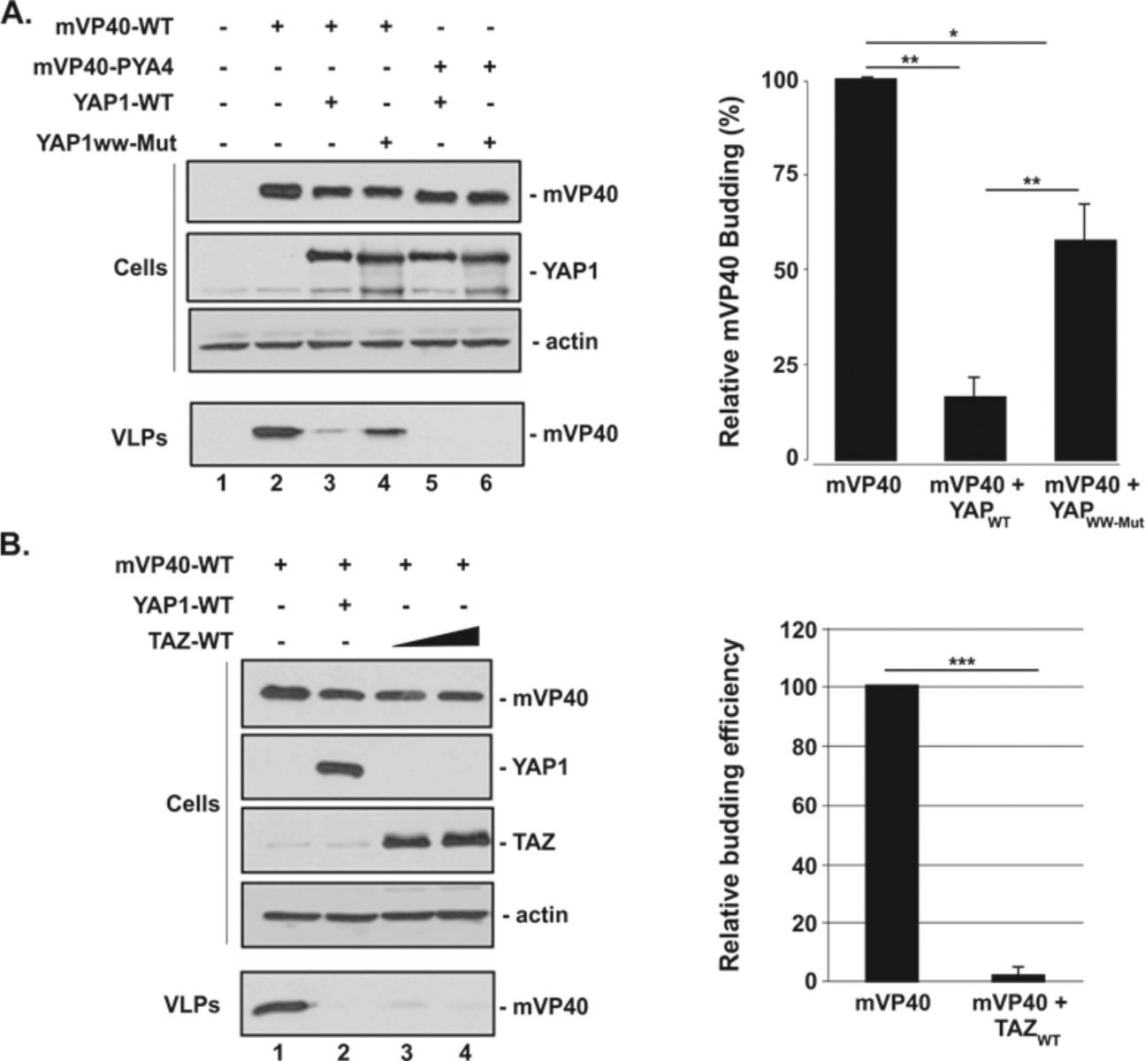
mVP40 VLP budding assay with YAP or TAZ. **A + B)** Budding of mVP40 VLPs was assessed from cells co-expressing WT or mutant forms of YAP or TAZ. HEK293T cells were transfected with the indicated combinations of plasmids (0.5μg/each for panel A, 0.25μg of mVP40-WT, YAP1-WT, and 0.25μg and 0.5μg of TAZ-WT for panel B), and both cell extracts and VLPs were harvested at 24 hrs post-transfection. The indicated proteins were detected and quantified in cells and VLPs by Western blotting and NIH Image-J, respectively. Bar graphs represent data from at least three independent experiments, and samples were compared using Welch’s t-test. * p<0.05, ** p<0.01, *** p<0.001.

Next, we sought to determine whether exogenous expression of either YAP1 or TAZ with mVP40 would affect egress of mVP40 VLPs. Briefly, mVP40-WT or budding defective mutant mVP40-PYA4 were co-expressed with YAP1 WT or a mutant lacking the WW-domain (YAP1 mut) in HEK293T cells, and both cell extracts and VLPs were harvested at 24 hours post-transfection and analyzed by Western blotting (Fig 2A). When equal amounts of mVP40-WT and YAP1 WT plasmids were co-transfected, we observed a consistent and significant decrease in mVP40 VLP egress (Fig 2A, compare lanes 2 + 3). Interestingly, co-expression of mVP40 with the YAP1 WW domain mutant did not yield the same level of VLP inhibition (Fig 2A, compare lanes 2 + 4). As expected, the mVP40-PYA4 mutant was defective in its ability to bud on its own, and thus there was no observed effect of YAP1 WT or mutant proteins on egress of mVP40-PYA4 (Fig 2A, lanes 5 + 6). Likewise, we observed a similar inhibitory effect on mVP40 VLP egress when TAZ-WT was co-expressed with mVP40 (Fig 2B). Together, these data show that either YAP1 or TAZ expression lead to inhibition of mVP40 VLP egress, which likely occurs in a WW-domain dependent manner.

### YAP1 overlaps with mVP40 in the cytoplasm and sequesters mVP40 away from the plasma membrane

**Fig 3:**
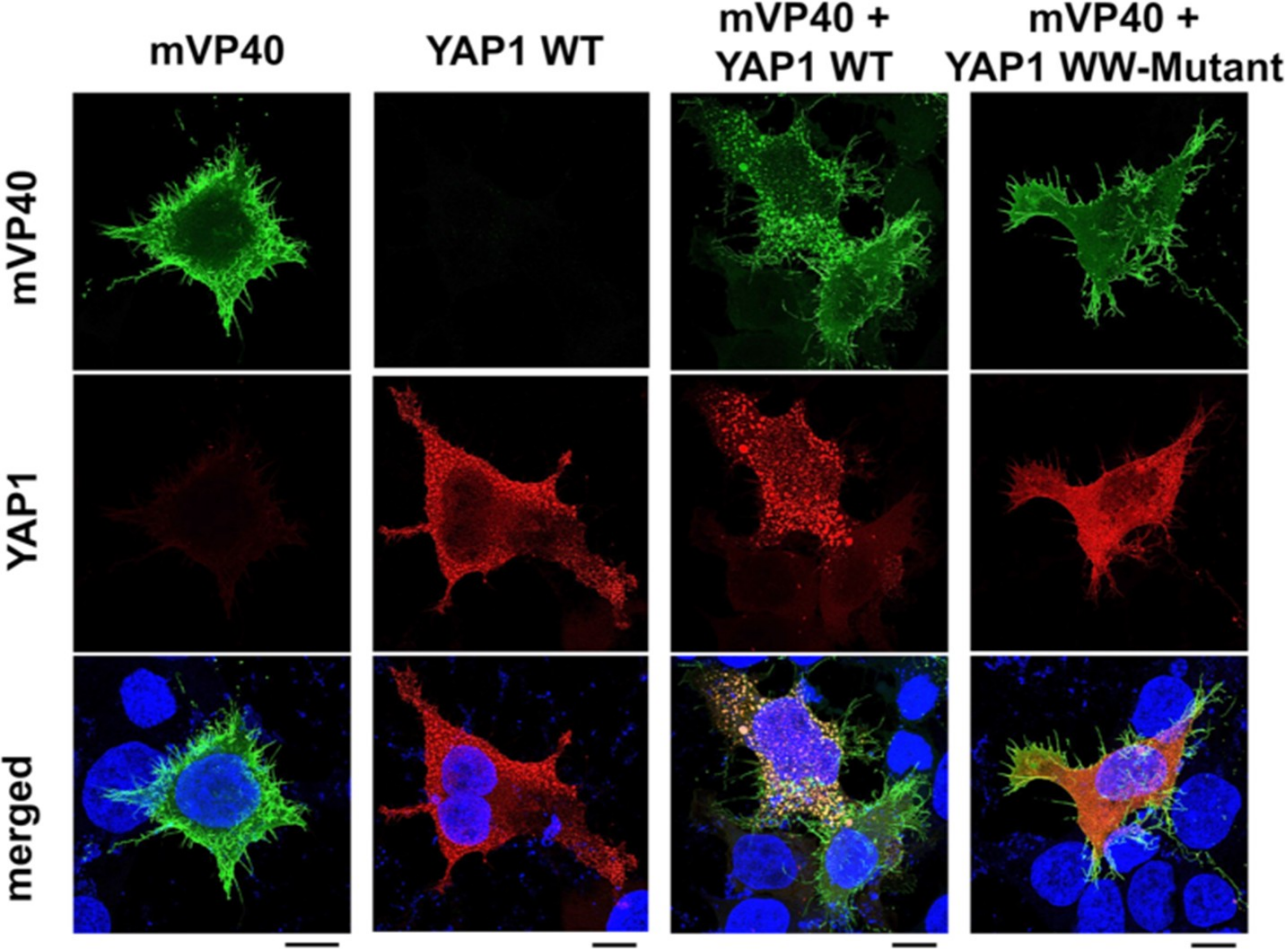
Intracellular localization of mVP40 and YAP. HEK293T cells were transfected with YFP-mVP40 alone, CFP-YAP1 WT alone, YFP-mVP40 + CFP-YAP1 WT, or YFP-mVP40 + CFP-YAP1 WW mutant (0.25μg/each). Confocal images for each condition are shown with mVP40 in green, YAP in red, and nuclei in blue. Overlap of YFP-mVP40 and CFP-YAP WT is indicated by yellow fluorescence in merged images. Scale bars = 10μm.

We used confocal microscopy to determine whether YAP1 WT or YAP1 WW-domain mutant exhibited a similar localization pattern with mVP40 in HEK293T cells. As expected, expression of mVP40 alone resulted in abundant localization at the cell surface and its appearance in membrane projections was indicative of efficient VLP egress (Fig 3, mVP40). Ectopic expression of YAP1 alone revealed a mainly cytoplasmic pattern of localization (Fig 3, YAP1 WT). When co-expressed, mVP40 overlapped with YAP1 WT in punctate aggregates in the cytoplasm and the appearance of membrane projections was greatly reduced (Fig 3, mVP40 + YAP1 WT). Interestingly, YAP1 WW-domain mutant did not overlap significantly with mVP40, and the mVP40 membrane projections remained prominent in these cells (Fig 3, mVP40 + YAP1 WW-mutant). Together, these data correlate well with VLP budding data above to suggest that ectopically expressed YAP1 interacts with mVP40 in the cytoplasm in a PPxY/WW-domain dependent manner to inhibit VLP egress, potentially by sequestering mVP40 away from the site of budding at the plasma membrane.

### Inhibition of mVP40 VLP egress is rescued by nuclear localization of YAP1

**Fig 4:**
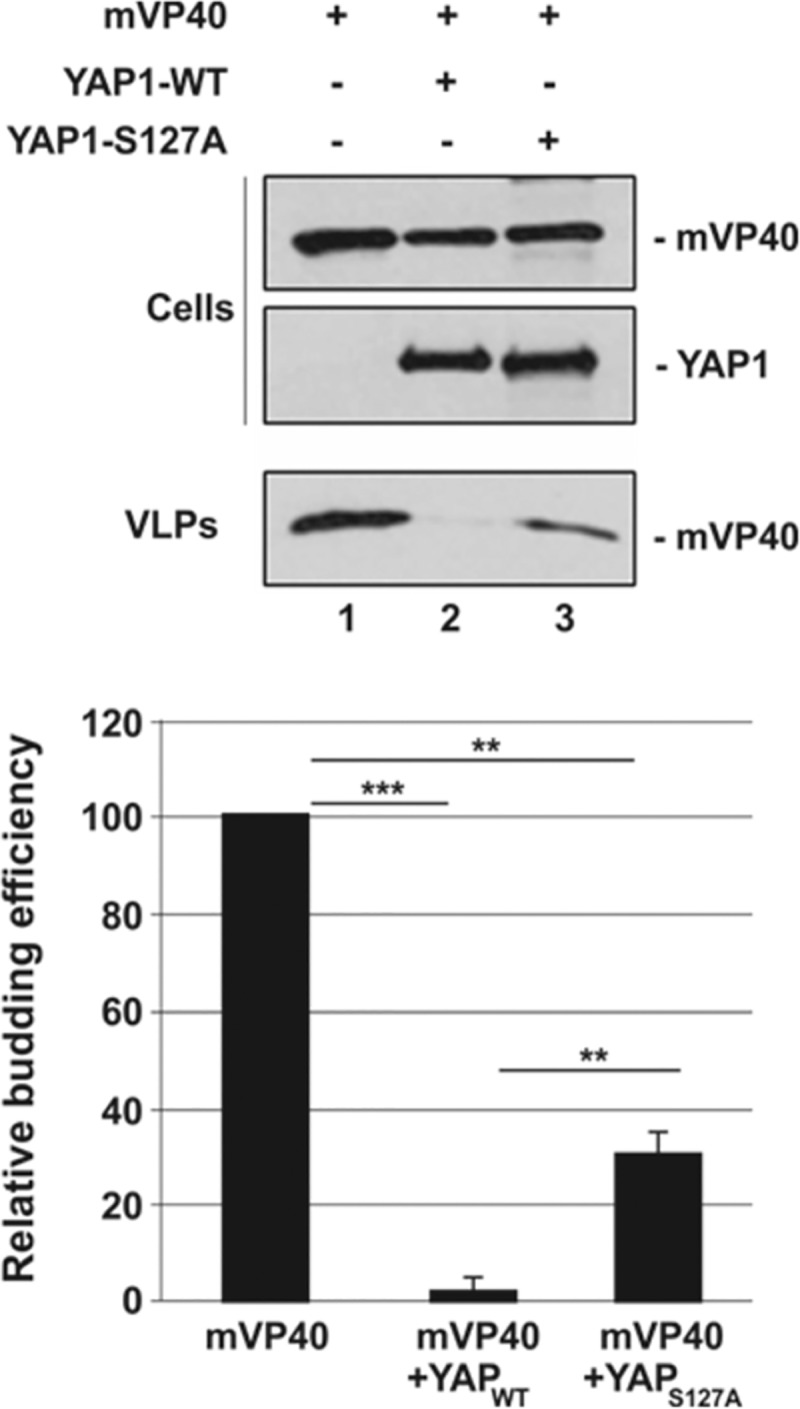
mVP40 VLP budding assay with WT and mutant YAP1. HEK293T cells were transfected with the indicated combinations of plasmids (0.25μg of mVP40 and 0.5μg of YAP plasmids), and both cell extracts and VLPs were harvested at 24 hrs post-transfection. The YAP1 S127A mutant cannot be phosphorylated at this position and is predominantly found in the nucleus. The indicated proteins were detected and quantified in cells and VLPs by Western blotting and NIH Image-J, respectively. Bar graphs represent data from at least three independent experiments. ** p<0.01, *** p<0.001.

The function of YAP1 as a transcriptional effector of the Hippo pathway is dependent on its phosphorylation state, which dictates its ability to either enter the nucleus (unphosphorylated) or remain sequestered in the cytoplasm (phosphorylated) [71]. Therefore, we next sought to determine whether the predominantly nuclear localization of exogenously expressed YAP1 would reduce its availability to interact directly with mVP40 resulting in an increase in VLP egress. Toward this end, we ectopically expressed YAP1-S127A, a well characterized, transcriptionally active mutant that is not phosphorylated on Serine127 and thus localizes predominantly to the nucleus [72,73]. Briefly, HEK293T cells were transfected with mVP40 alone, mVP40 plus YAP1 WT, or mVP40 + YAP1 S127A, and both cell extracts and VLPs were harvested and analyzed by Western blotting (Fig 4). As described above, expression of YAP1 WT resulted in inhibition of mVP40 VLP egress; however, the degree of inhibition was significantly reduced in the presence of mutant YAP1 S127A in repeated experiments (Fig 4, compare lanes 2 + 3). These results suggest that when YAP1 is largely in the cytoplasm, its ability to engage mVP40 results in inhibition of VLP egress, whereas when YAP1 is predominantly in the nucleus, it cannot engage mVP40 as efficiently and thus has less of an impact on VLP egress.

### Budding of mVP40 VLPs is enhanced in the context of endogenous Hippo pathway activation

**Fig 5:**
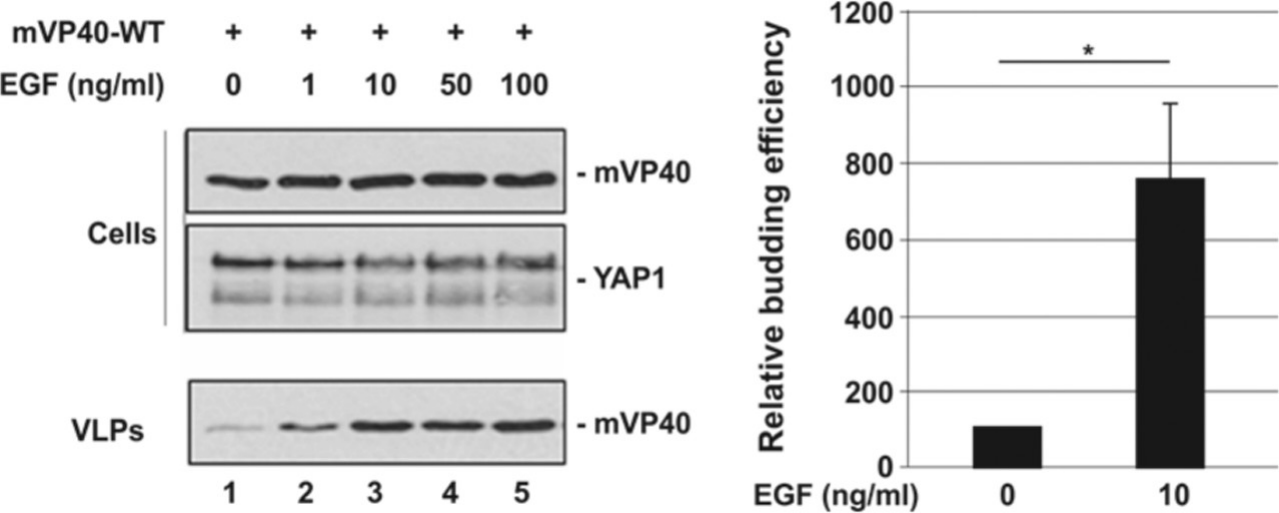
Activation of the Endogenous Hippo pathway impacts mVP40 VLP budding. U2OS cells were transfected with mVP40-WT (2μg) for 24 hrs, and then stimulated for an additional 24 hrs with the indicated concentration of purified EGF. The indicated proteins were detected and quantified in cells and VLPs by Western blotting and NIH Image-J, respectively. The bar graph represents data from three independent experiments of cells stimulated with either 0 or 10 ng/ml of EGF. The relative budding efficiency of mVP40 peaked at 10ng/ml of EGF with an average increase of approximately 7.5-fold vs. control. * p = 0.03 as determined by Welch’s t-test.

We next sought to determine whether mVP40 VLP budding would be impacted following activation of the endogenous Hippo pathway by treating mVP40-expressing cells with epidermal growth factor (EGF). Briefly, U2OS cells were first transfected overnight with mVP40-WT, and then treated with increasing amounts of EGF, which has been shown to trigger translocation of endogenous YAP into the nucleus [74]. We observed a dose-dependent and significant increase (up to 7.5-fold) in mVP40 VLP egress in the presence of increasing concentrations of EGF (Fig 5, lanes 1–5). Cellular levels of mVP40 and endogenous YAP1 were found to be equivalent in all samples (Fig 5, lanes 1–5). These results are consistent with those shown in Fig 4 and suggest that endogenous Hippo pathway activation regulates mVP40-mediated egress.

### Budding of mVP40 VLPs occurs efficiently in YAP knockout (KO) cells

**Fig 6:**
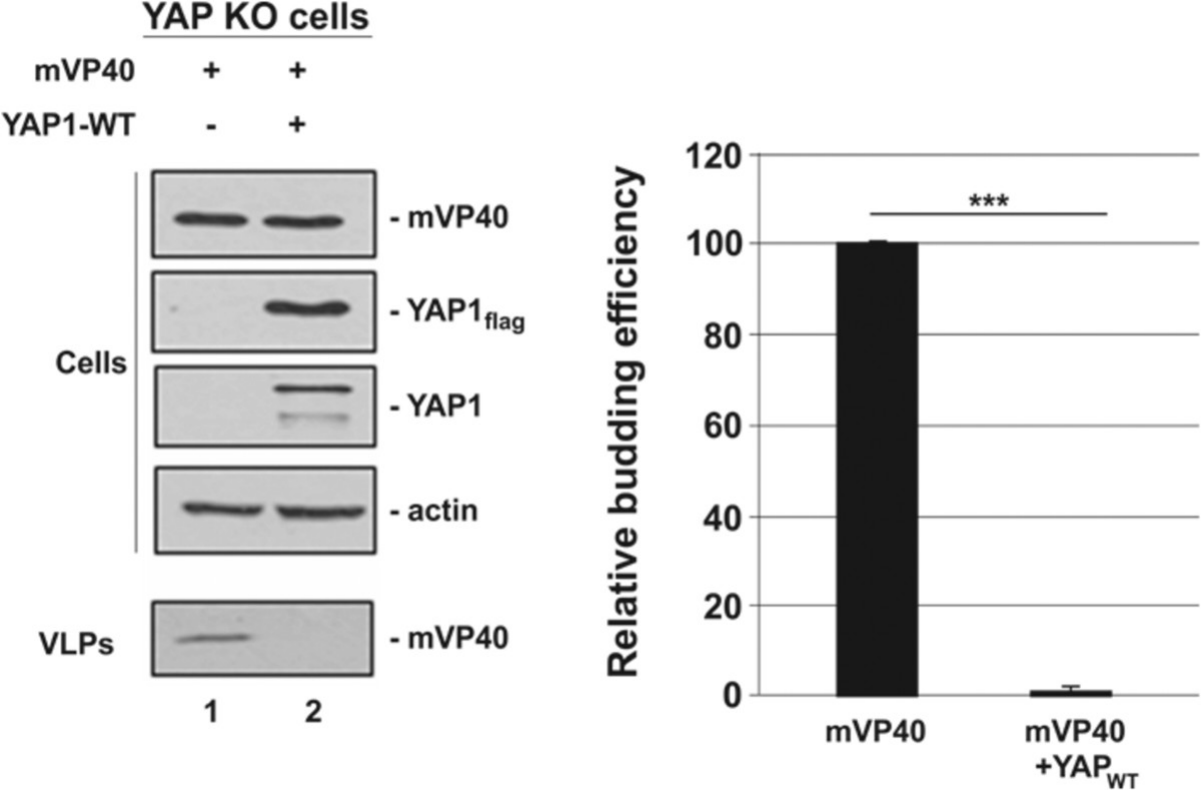
mVP40 VLP budding assay in YAP knockout cells. YAP knockout (KO) cells were transfected with the indicated combinations of plasmids (0.25μg/each), and both cell extracts and VLPs were harvested at 24 hrs post-transfection. The indicated proteins were detected and quantified in cells and VLPs by Western blotting and NIH Image-J, respectively. Bar graph represents data from at least three independent experiments. *** p<0.001.

To further validate the role of YAP as a potential regulator of mVP40 VLP egress, we used an HEK293-based YAP KO cell line [75]. Briefly, YAP KO cells were transfected with either mVP40 alone or mVP40 + YAP1 WT, and both cell extracts and VLPs were harvested and analyzed by Western blotting (Fig 6). We observed efficient egress of mVP40 VLPs from the YAP KO cells; however, mVP40 VLP egress was significantly inhibited when the YAP KO cells were spiked with exogenous YAP1 WT (Fig 6). These results are consistent with those described above, and taken together, the results shown in Figs 2–6 suggest that YAP/TAZ and the Hippo pathway have a functional impact on mVP40-mediated budding.

### mVP40 VLP budding is inhibited in shAmot knockdown cells

**Fig 7:**
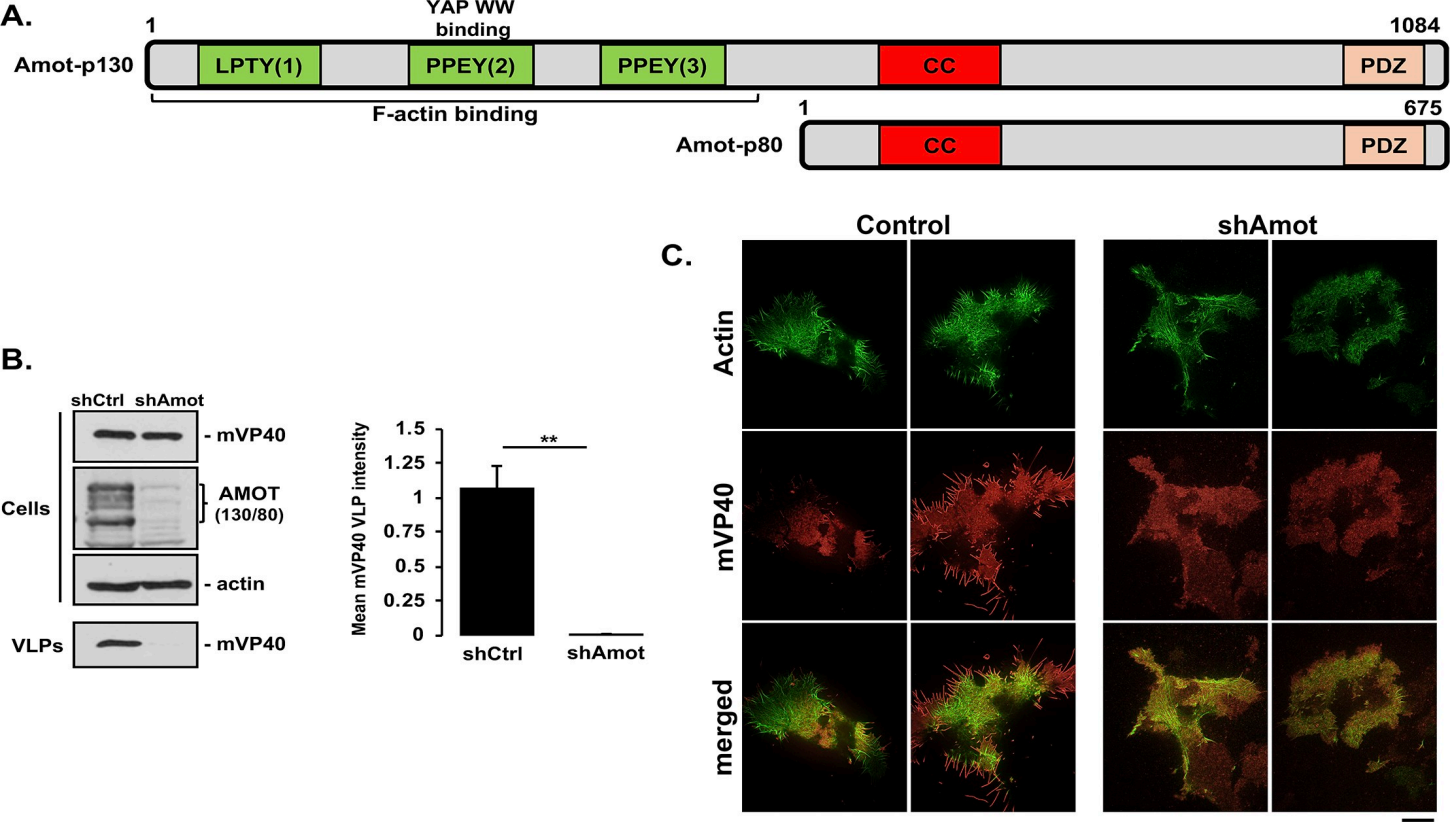
VP40 VLP budding assay in AMOT control (shCtrl) or AMOT knockdown (shAmot) cells. **A)** Schematic diagrams of full-length angiomotin (Amot-p130) and N-terminally truncated angiomotin (Amot-p80) with key domains highlighted. Numbers represent amino acids. LPTY(1), PPEY(2), and PPEY(3) = three PPxY motifs; CC = coiled coil domain; PDZ = PSD-95/Dlg1/ZO-1 domain. **B)** AMOT expressing (shCtrl) or knockdown (shAmot) cells were transfected with mVP40 (0.25μg) and the indicated proteins were quantified in cells and VLPs by Western blotting and NIH Image-J, respectively. The bar graph represents data from at least three independent experiments. ** p<0.01. **C)** shCtrl or shAmot cells were transfected with 0.5μg each of pCMV-LifeAct-RFP and YFP-mVP40 plasmids, and cells were imaged by TIRF microscopy at 24 hours post-transfection. Two representative images of F-actin alone (green), YFP-mVP40 alone (red), and merged images for both shCtrl and shAmot cells are shown. Scale bar = 10 μm.

Human angiomotin (Amot-p130) contains multiple PPxY motifs at its N-terminus and is a well-characterized, strong interactor and regulator of YAP1 localization and activity, whereas Amot-p80 is an N-terminally truncated mutant lacking all PPxY motifs (Fig 7A) [61,63–65,69,76,77]. This prompted us to ask whether Amot expression would have an effect on mVP40 VLP egress. We used an HEK293T-based cell line stably expressing a lentiviral short hairpin RNA specific for Amot (shAmot) to assess egress of mVP40 VLPs compared to that from Amot expressing control cells (shCtrl) [64]. Briefly, shCtrl or shAmot cells were transfected with mVP40 (Fig 7B), and both cell extracts and VLPs were harvested and analyzed by Western blotting. Indeed, we observed robust inhibition of mVP40 VLP egress in the shAmot cells compared to egress of mVP40 VLPs in shCtrl cells (Fig 7B). Since budding of mVP40 VLPs was reduced significantly in shAmot cells, we sought to determine whether the mechanism of inhibition correlated with Amot’s role as an F-actin binding protein. Toward this end, we co-transfected either shCtrl or shAmot cells with plasmids fluorescently labeling F-actin (pCMV-LifeAct-RFP) and YFP-mVP40, and used TIRF microscopy to visualize both F-actin and mVP40 localization at the plasma membrane (Fig 7C). We observed robust localization of both F-actin and mVP40 in well-formed, prominent surface membrane projections in shCtrl cells. Interestingly, F-actin was most prominent at the bases of the membrane projections, whereas mVP40 was observed throughout the projections (Fig 7C). In contrast, shAmot cells displayed significantly fewer membrane projections than shCtrl cells, and the localization patterns of both F-actin and mVP40 appeared more diffuse and disorganized (Fig 7C). Together, these results not only suggest that expression of endogenous Amot is important for mVP40 VLP formation/protrusion at the plasma membrane and subsequent egress, but also that the mechanism by which Amot regulates VLP formation and egress may be linked to its role in binding and organizing F-actin filaments at the cell surface.

### Release and spread of authentic MARV is inhibited in shAmot knockdown cells

**Fig 8:**
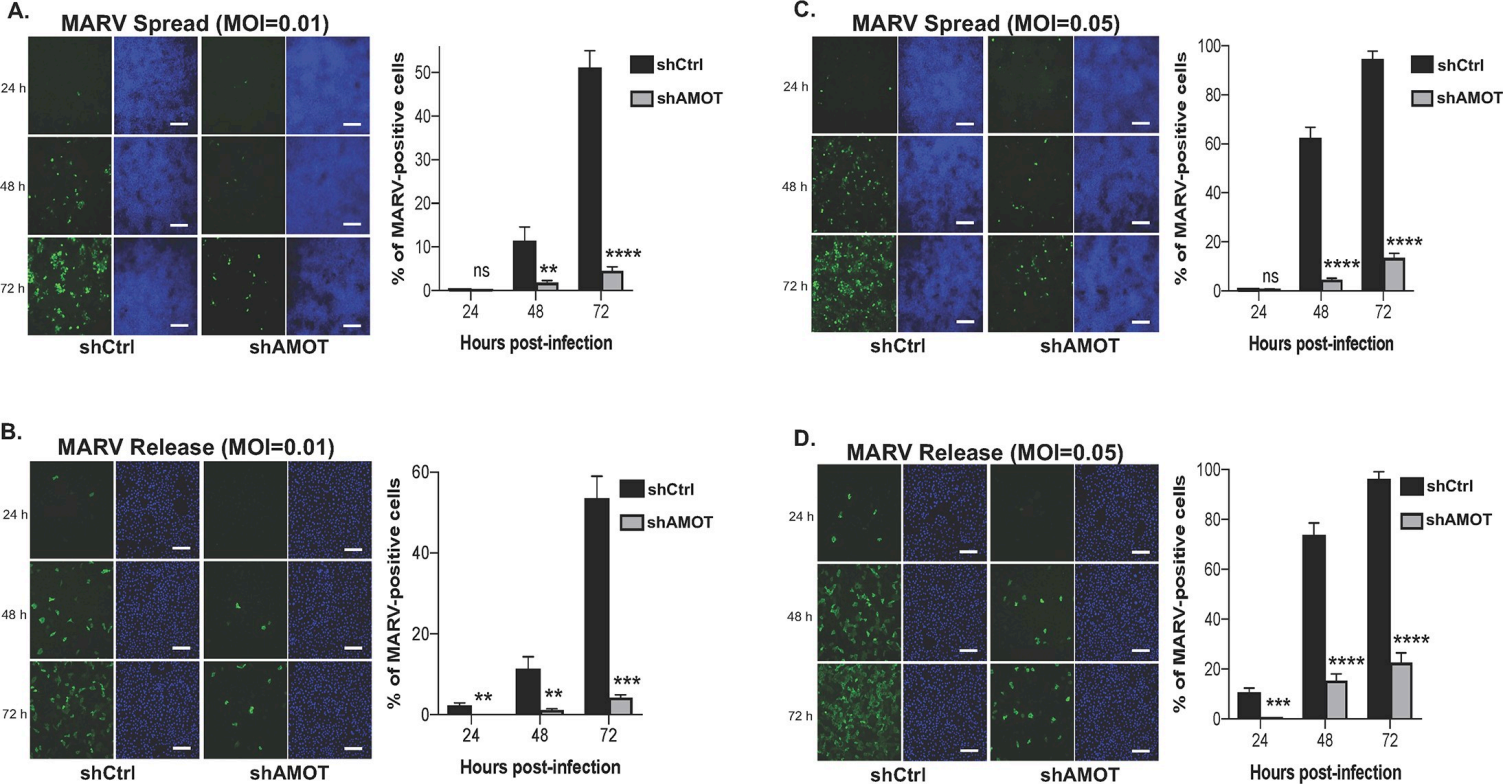
Spread and release of live MARV from shCtrl or shAmot cells. shCtrl or shAmot cells were infected with MARV at an MOI of either 0.01 (**A** and **B**), or 0.05 (**C** and **D**), and both virus spread (**A** and **C**) and release (**B** and **D**) were quantified at 24, 48, and 72 hours post-infection. MARV = green, Hoechst 33342-stained nuclei = blue. Bar graphs represent data from three independent experiments. Scale bars = 200 μm.

Next, we asked whether budding and spread of authentic MARV strain Musoke would also be inhibited in the shAmot cells compared to that in shCtrl cells. Briefly, shCtrl or shAmot cells in 96-well plates were infected with MARV strain Musoke (Fig 8) [78] at MOIs of 0.01 or 0.05, and the cells were fixed at 24, 48, and 72 hours post-infection to assess virus spreading. Indeed, spread of MARV throughout the cultures was inhibited significantly in the shAmot cells compared to that observed in the shCtrl cells (Fig 8A and 8C). Similarly, release of infectious MARV was also inhibited significantly in the shAmot cells compared to that observed in the shCtrl cells (Fig 8B and 8D). Taken together, we observed that expression of endogenous Amot was crucial not only for VP40 VLP egress, but also for spread and egress of infectious filoviruses.

### Expression of Amot overcomes YAP1-mediated inhibition of mVP40 VLP budding

**Fig 9:**
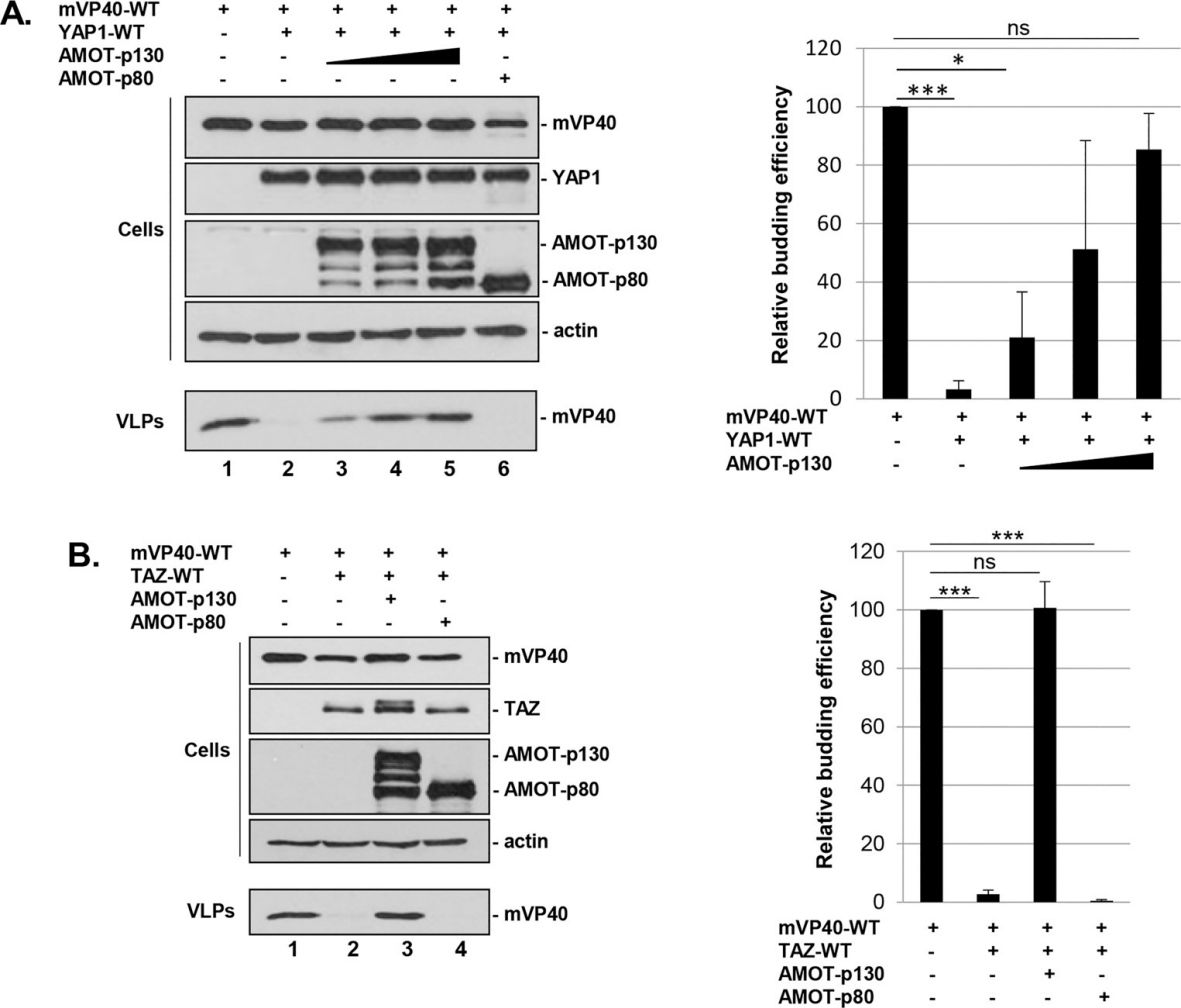
Angiomotin rescues budding of mVP40 VLPs in the presence of YAP or TAZ. **A + B)** HEK293T cells were transfected with the indicated combinations of plasmids (0.25μg of mVP40, 0.25μg/each of YAP1-WT and TAZ-WT, 0.25μg, 0.5μg or 1.0μg of Amot-p130, and 1.0μg of Amot-p80) and both cell extracts and VLPs were harvested at 24 hrs post-transfection. The indicated proteins were detected and quantified in cells and VLPs by Western blotting and NIH Image-J, respectively. Bar graphs represent data from at least three independent experiments. **A)** * p<0.05, *** p<0.001, ns = not significant; **B)** *** p<0.001, ns = not significant.

Since the PPxY motifs of Amot interact strongly with the WW-domain of YAP1, we wanted to determine whether Amot-p130 could compete in a PPxY-dependent manner with mVP40 for binding to YAP1 and thus relieve the inhibitory effect of YAP1 on mVP40 VLP egress. Toward this end, HEK293T cells were transfected with the indicated combinations of plasmids (Fig 9A), and both cell extracts and VLPs were harvested and analyzed by Western blotting. As before, expression of YAP1 WT significantly inhibited egress of mVP40 VLPs (Fig 9A, compare lanes 1 + 2); however, upon co-expression of increasing amounts of Amot-p130, budding of mVP40 VLPs was rescued to near WT levels (Fig 9A, lanes 3–5). Importantly, mVP40 VLP budding was not rescued in the presence of Amot-p80, which lacks the PPxY motifs (Fig 9A, lane 6). These results were identical to those observed with TAZ-WT plus either Amot-p130 or Amot-p80 (Fig 9B). Together, these results show that ectopic expression of Amot-p130 was able to rescue budding of mVP40 VLPs in the presence of either YAP1 WT or TAZ-WT in a PPxY-dependent manner, and suggest that the competitive interplay among Amot, YAP1, TAZ, and mVP40 may be mechanistically important for either positive or negative regulation of mVP40-mediated egress.

### Amot-p130 PPxY motifs 2 and 3 are important for rescue of mVP40 VLP budding

**Fig 10:**
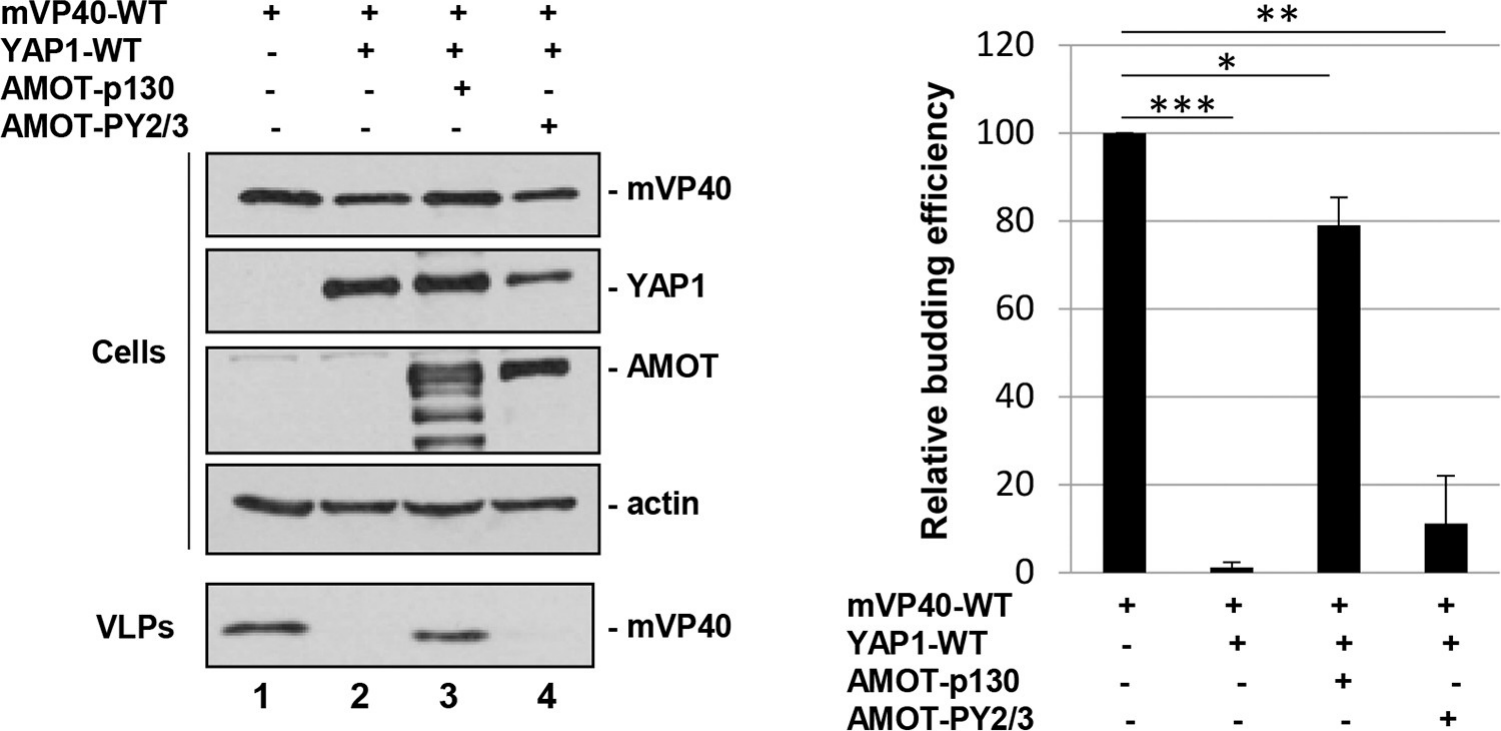
Angiomotin rescue of mVP40 VLP budding is dependent on Amot’s PPxY motifs. HEK293T cells were transfected with the indicated combinations of plasmids (0.25μg of mVP40, 0.25μg of YAP1-WT, 1.0μg of Amot-p130, and 1.0μg of Amot-PY2/3), and both cell extracts and VLPs were harvested at 24 hrs post-transfection. Amot-PY2/3 contains mutations in PPEY motifs #2 and #3 (see Fig 6A). The indicated proteins were detected and quantified in cells and VLPs by Western blotting and NIH Image-J, respectively. Bar graph represents data from at least three independent experiments. * p<0.05, ** p<0.01, *** p<0.001.

To more precisely determine whether the PPxY motifs of Amot-p130 were necessary for rescue of mVP40 VLP budding in the presence of YAP1, we used a double PPxY mutant of Amot-p130 in which PPxY motifs 2 and 3 have been mutated [68]. We chose this mutant since PPxY motifs 2 and 3 of Amot-p130 are more conventional and match more closely to the PPxY core motif conserved in mVP40 (Fig 7A). Briefly, HEK293T cells were transfected with the indicated combinations of plasmids (Fig 10), and both cell extracts and VLPs were harvested and analyzed by Western blotting. As we observed previously, YAP1 WT inhibited mVP40 VLP egress (Fig 10, lane 2), whereas the addition of an equal amount of Amot-p130 overcame this inhibition to rescue budding of mVP40 VLPs (Fig 10, lane 3). Interestingly, expression of mutant Amot-PY2/3 did not rescue budding of mVP40 VLPs, suggesting that either one or both of these PPxY motifs of Amot-p130 are critical for VLP rescue, again likely due to competition for binding to WW1 domain of YAP1.

### Filoviral and host PPxY motifs can bind to WW1 domain of YAP

**Fig 11:**
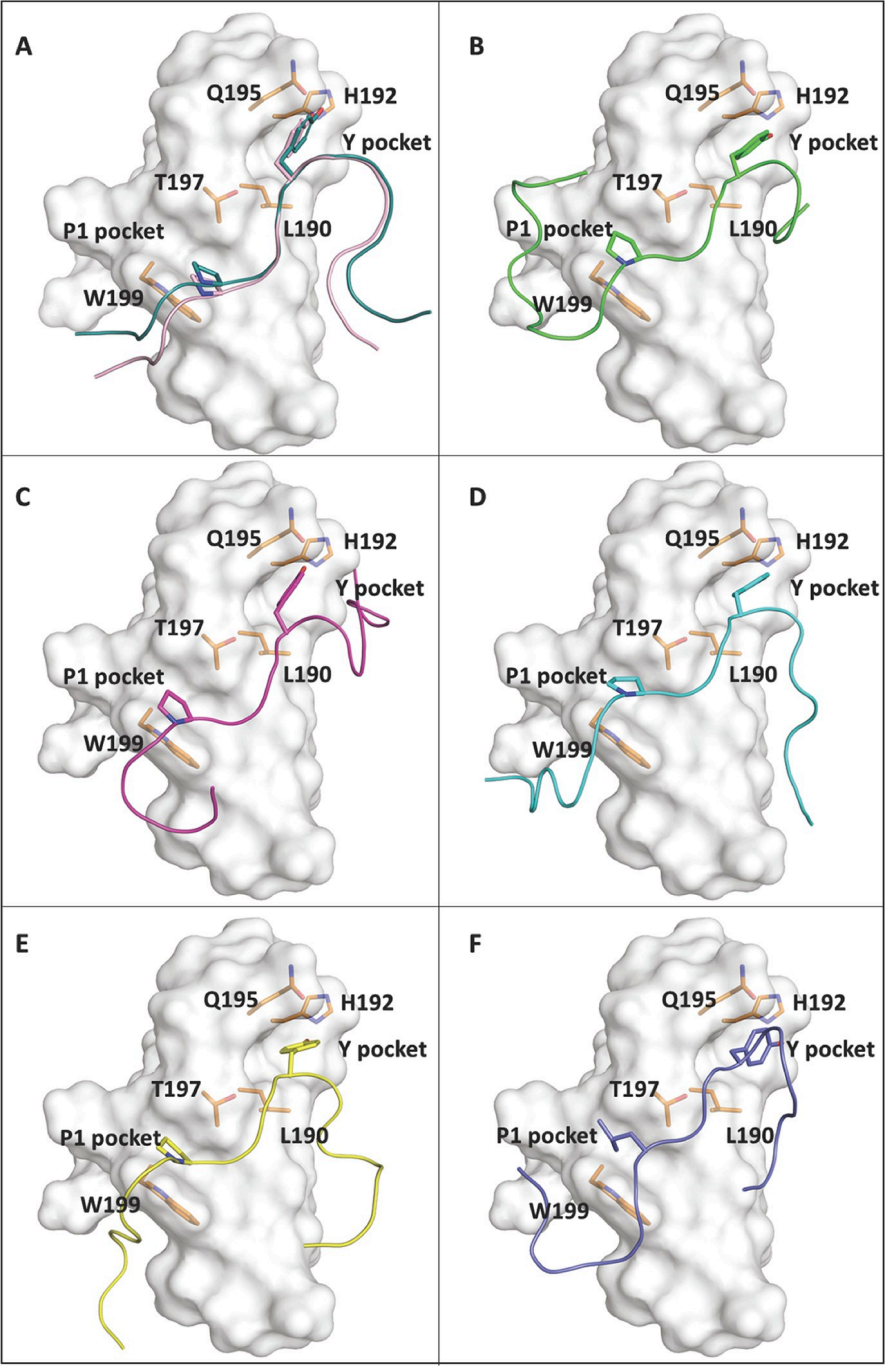
Protein-peptide docking poses. The protein-peptide docking poses are shown for: **A)** the NMR structure of YAP WW1-smad7 peptide (GESP**PPPY**SRYPMD) complex (light pink color) and the docking pose of smad7 peptide (control, deep teal color), **B)** YAP WW1 domain-eVP40 peptide (MRRVILPTA**PPEY**MEAI) (green), **C)** YAP WW1 domain-mVP40 peptide (MQYLN**PPPY**ADHGANQL) (magenta), **D)** YAP WW1 domain-Amot peptide 1 (GMEHRGP**PPEY**PFKGMP) (cyan), **E)** YAP WW1 domain-Amot peptide 2 (QLMRYQH**PPEY**GAARPA) (yellow), and **F)** YAP WW1 domain-Amot peptide 3 (MQNNEE**LPTY**EEAKVQ) (purple). The first Proline (P1) of the PPxY motif (or L of LPTY in Amot peptide 3) occupies the P1 pocket of YAP WW1 domain which is formed by T197 and W199, and the Y sidechain of the PPxY motif occupies the Y pocket (hydrophobic groove composed by L190, H192, Q195, and T197) of YAP WW1 domain.

To complement the biochemical experiments described above, we sought to use a structure-based docking approach to assess the binding potential of the filoviral VP40 and Amot PPxY containing peptides to interact with YAP WW1 domain (Fig 11). The YAP WW domains exhibit a typical triple-stranded anti-parallel *β*-sheet structure, with the glycine linker forming an unstructured loop that holds the binding peptide in close proximity to the domain. PPxY peptides exhibit a polyproline type II (PPII) helical conformation. There are two pockets that recognize the PPxY peptide; the P1 pocket is formed by the side chains of W199 and T197 and accommodates P1, and the Y pocket is a hydrophobic groove composed of sidechains from L190, H192, Q195, and T197 that fit the Y sidechain of the PPxY peptide (Fig 11).

To validate our Glide peptide docking module, we first docked the Smad7 peptide to the YAP WW1 domain. Our ability to reproduce the crystal structure of the smad7 peptide (RMSD 1.2 Å) (Fig 11A) confirmed its reliability. Next, we tested both eVP40 and mVP40 PPxY peptides as well as the three Amot-p130 PPxY peptides for their ability to dock with the PPxY binding region of YAP WW1 domain (Fig 11, panels B-F). We found that all five peptides could bind to the PPxY binding pocket of YAP WW1 domain; however, the mVP40 PPxY peptide yielded the best docking score (Emodel score of -166.8). Indeed, the tyrosine (Y) residue found before the PPxY motif in the mVP40 and the histidine (H) residue following the PPxY motif in the mVP40 peptide mediated direct interactions with the YAP WW1 domain and contributed to the strength of binding. Following the mVP40 PPxY peptide, the next strongest interactions were mediated by Amot-2, Amot-1, eVP40, and Amot-3 peptides. These observations support the potential for competition between filoviral and host PPxY motifs for binding to YAP WW1 domain, which not only may regulate viral egress and transmission, but also may affect the localization of YAP1 and thus its function as the major effector of the Hippo pathway.

## Discussion

The PPxY L-domain motifs of EBOV and MARV VP40 interact with specific host proteins that contain one or more modular WW-domains to regulate VLP/virus assembly, egress, and spread [9,10,17,79]. As the PPxY core motif is physically and functionally conserved in a wide array of matrix proteins encoded by viruses belonging to the *Filoviridae*, *Arenaviridae*, *Retroviridae*, *Rhabdoviridae*, *and Paramyxoviridae* families [16,80], a better understanding of the breadth and function of PPxY-dependent host interactions during the budding process will likely have wide-ranging implications for the biology and pathogenesis of a plethora of viral pathogens.

Here we identified two novel mVP40 PPxY interactors, YAP1 and TAZ, that in addition to the recently identified host interactor BAG3 [81,82], are members of a growing list of WW-domain bearing host proteins that negatively regulate VP40-mediated egress. Expression of either WT YAP1 or WT TAZ led to a significant decrease in mVP40 VLP egress. Moreover, budding of mVP40 VLPs was efficient in YAP KO cells; however, upon expression of exogenous YAP1, VLP budding decreased significantly. While the identity and number of negative regulators of budding continues to expand, the majority of previously characterized host PPxY interactors positively affect egress (*e*.*g*. members of the HECT family of E3 ubiquitin ligases). We speculate that viruses such as EBOV and MARV have evolved mechanisms to mimic the modular nature of host interactions to hijack or recruit specific host proteins to facilitate or enhance virus egress and spread. Conversely, we envision host cells countering with innate defense mechanisms that target these conserved viral motifs to inhibit or disrupt virion assembly and egress. For example, BAG3 is a co-chaperone and cell survival protein recently shown to interact with the mVP40 and eVP40 PPxY motifs to sequester VP40 in the cytoplasm, away from the site of budding at the plasma membrane [81,82]. Interestingly, the BAG3 WW-domain has been reported to engage in the PPxY/WW-domain interactions that regulate YAP/TAZ signaling, and as a result, BAG3 may modulate YAP/TAZ-mediated co-transcriptional activity of genes involved in cell proliferation, actin dynamics and crosslinking, and cell adhesion/migration [83].

As the downstream effector of the Hippo pathway, YAP can either be sequestered in the cytoplasm in a phosphorylated form (Hippo ON), or shuttled into the nucleus in an unphosphorylated form (Hippo OFF), where it will engage TEAD transcription factors to upregulate genes involved in cell proliferation. We found that ectopic expression of a phosphorylation mutant of YAP1 (localized predominantly in the nucleus) partially rescued egress of mVP40 VLPs (Fig 4), likely due in part to the inability of nuclear YAP1 to physically engage mVP40 and sequester it away from the plasma membrane. In addition, treatment of mVP40-expressing cells with EGF to trigger translocation of endogenous YAP1 into the nucleus, resulted in a dose-dependent increase in VLP budding (Fig 5). It is also tempting to speculate that once YAP1 enters the nucleus, upregulation of genes involved in cell proliferation/migration, actin polymerization, and filopodia formation may also promote an intracellular environment that is favorable for efficient VLP formation and/or egress. These studies, as well as those using authentic filoviruses, will be necessary to demonstrate a role for the endogenous Hippo pathway in impacting filovirus replication.

One of the key regulators of YAP1 localization and activity is Amot, which binds strongly to the first WW-domain of YAP1 *via* one of its multiple N-terminal PPxY motifs [84]. Amot, along with other family members, plays an important role in the biology of the vascular system, and Amot has been linked previously to budding of other RNA viruses [85–88]. Interestingly, we observed a dose-dependent rescue of mVP40 VLP budding when increasing amounts of Amot were added to cells co-expressing mVP40 and either YAP1 or TAZ. Moreover, we used shCtrl (expressing endogenous Amot) and an shAmot knockdown cell line to show that endogenous Amot was critical for efficient egress of mVP40 VLPs (Fig 7B). Images from TIRF microcopy provided mechanistic insights into the observed Amot-mediated changes in VLP egress by revealing significantly distinct patterns of localization of both F-actin filaments and mVP40 expressed at the plasma membrane of either shCtrl or shAmot cells (Fig 7C). Indeed, these findings revealed that the formation and organization of F-actin filaments sharply aligned with mVP40 at the bases of the visible VLP membrane projections in shCtrl cells, whereas this alignment and organization of F-actin with mVP40 VLPs was largely absent in shAmot cells (Fig 7C). Importantly, we found that egress and spread of live MARV (Fig 7) was also significantly more robust in shCtrl cells expressing endogenous Amot than that observed in shAmot knockdown cells at 24, 48, and 72 hours post-infection. The ability of Amot to rescue budding of mVP40 VLPs in the presence of YAP1 was likely due to competition between the PPxY motifs of Amot and those of mVP40 for binding to endogenous YAP1. Indeed, Amot-p80, which lacks all of the N-terminal PPxY motifs, was unable to rescue mVP40 VLP budding, and we demonstrated that PPxY motifs 2 and 3 of Amot-p130 were critical for rescue of mVP40 VLP egress. Additionally, the well-documented physical interaction of Amot with LATS1/2 kinases, which results in the activation of the kinases and sequestration of phosphorylated YAP1 and TAZ proteins in the cytoplasm, deserves careful study in the context of VP40 signalling [76].

**Fig 12:**
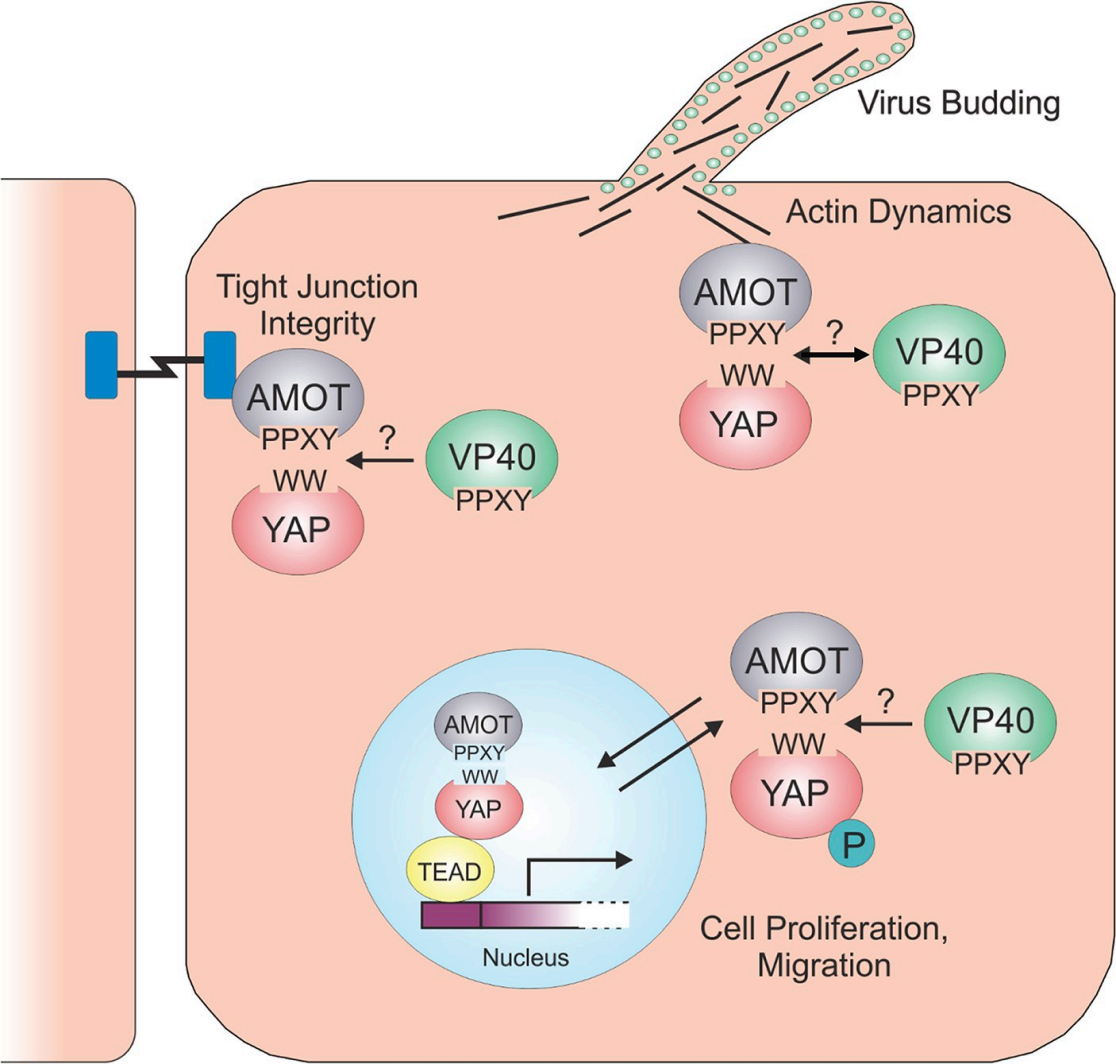
Working model of modular competition between filovirus VP40 and Hippo pathway components YAP and AMOT. Potential competition (?) between the PPxY motifs of filovirus VP40 and those of AMOT for binding to WW-domains of YAP (and/or TAZ) may affect cellular processes including, actin dynamics and filopodia formation, tight junction formation and integrity, and upregulation of genes involved in cell proliferation and migration.

Amot binds and sequesters YAP1 on both actin filaments and at cellular tight junctions (Fig 12) [59,60,62,64,65,89–94]. The Amot/YAP1 complex may also enter the nucleus leading to upregulation of a set of genes that may be distinct from those upregulated by YAP1 alone [61,64,66]. Thus, it is possible that modular competition between PPxY motifs of filoviral VP40 and Amot for binding to the WW domain of YAP1 could impact several processes in the host cell including: 1) actin dynamics and polymerization, 2) tight junction formation and integrity, and 3) upregulation of cell proliferation genes (Fig 12). Our data indicate that competition for binding among YAP1/Amot/mVP40 complexes, as well as Amot’s role in regulating F-actin dynamics at the plasma membrane [62,90], may both play a mechanistic role in impacting VP40-mediated egress.

Lastly, filovirus infection could potentially influence Hippo signaling. For example, if VP40 competes with Amot for binding and/or sequestering YAP1 in the cytoplasm, this may affect YAP1-regulated genes and impact filovirus egress and spread. Indeed, several YAP1-responsive genes encode proteins involved in actin polymerization, filopodia formation, and plasma membrane projections involved in cell migration. Such outcomes would be beneficial for maximal levels of filovirus budding at the plasma membrane. Studies are now underway to identify and quantify both up- and down-regulated genes in cells expressing various combination of YAP1/Amot/VP40 to determine whether nuclear localized and transcriptionally active YAP1 regulates VP40-mediated egress. These findings may provide new insights into how potentially altering Hippo signaling pharmaceutically could perhaps impact virus budding, spread, and pathogenesis.

In sum, we identified YAP1 and TAZ as two novel WW-domain interactors with the PPxY motif of mVP40, that like BAG3, inhibited mVP40-mediated egress in a PPxY/WW-domain dependent manner. These findings are the first to link key effectors of the Hippo pathway to the biology and perhaps pathogenesis of filoviruses. These findings provide important insights into the roles of novel host proteins and a new cellular pathway in regulating filovirus VP40-mediated egress. The interplay among VP40/YAP1/TAZ/Amot may have wide ranging biological and pathological implications in filovirus infection and disease, and thus a more comprehensive understanding of these virus-host interactions may be helpful in the development of future antiviral therapies. Moreover, since many additional RNA viruses also contain PPxY L-domain motifs, these findings may implicate the Hippo pathway in the biology and egress of a wide array of viral pathogens.

## Materials and methods

### Cell lines and antisera

HEK293T, HEK293A YAP knockout cells (kindly provided by K-L. Guan, University of California, San Diego), HEK293T-based shCtrl, and shAmot cells (kindly provided by J. Kissil, Scripps Research Institute, FL) were maintained in Dulbecco’s modified Eagle’s medium (DMEM) supplemented with 10% fetal calf serum (FCS), penicillin (100 U/ml)/streptomycin(100μg/ml) at 37°C in a humidified 5% CO_2_ incubator [64,75]. Antisera used include: anti-FLAG antibody (Fitzgerald, CAT# 10R-7750) to detect ectopically expressed Flag-tagged mVP40 and Flag-tagged YAP1; anti-YAP1 antibody (clone 1A12, Cell signaling, CAT# 12395S); anti-ß-actin (Gene Tex, CAT# GT5512); anti-GST (Sigma); anti-Amot (EMD Millipore); anti-HA to detect HA-tagged TAZ (Sigma); anti-rabbit IgG-HRP (GE Healthcare, CAT# NA934V); or anti-mouse IgG-HRP (GE Healthcare, CAT# NA931V).

### Expression and Purification of GST-YAP1 WW fusion proteins

Expression and purification of GST fusion proteins from pGEX-YAP-WW1 and pGEX-YAP-WW2 plasmids (kindly provided by M. Bedford, Univ. of Texas) were performed as described previously [95]. Briefly, pGEX-YAP-WW1 and pGEX-YAP-WW2 plasmids were transformed into *E*. *coli* BL21(DE3) cells and single colonies were cultured in 10ml of LB media overnight with shaking at 37°C. The overnight culture was added into 100ml of fresh LB broth and grown at 37°C for one hour with shaking. GST-WW domain fusion proteins were induced with isopropyl-β-d-thiogalactopyranoside (IPTG) (0.1 mM) for 4 h at 30°C. Bacterial cultures were centrifuged at 5,000 rpm for 10 min at 4°C, and lysates were extracted by using B-PER bacterial protein extract reagent according to the protocol supplied by the manufacturer (Pierce). GST-YAP-WW1, GST-YAP-WW2, and GST-TAZ-WW domain fusion proteins were purified with glutathione-Sepharose 4B and eluted with elution buffer (100 mM Tris-Cl [pH 8.0], 120 mM NaCl, 30 mM reduced glutathione). Purified proteins were analyzed on SDS-PAGE gels and stained with Coomassie blue.

### GST-WW/mVP40 pulldown assay

GST-YAP-WW1, GST-YAP-WW2, and GST-TAZ-WW domain fusion proteins (10μg) were incubated with glutathione-Sepharose 4B beads in 500 μl of nonionic detergent-based solution in B-PER bacterial protein extract reagent kit overnight at 4°C. The glutathione-Sepharose 4B beads were washed 3 times with 1x PBS and then incubated with cell extracts from mock or mVP40 transfected HEK293T cells overnight at 4°C. The beads were then washed 5x with 1x mild buffer and suspended in 30μl of 2x loading buffer with boiling. Flag-tagged mVP40 protein was detected by SDS-PAGE and Western blotting with anti-flag antibody.

### Confocal microscopy

HEK293T cells on glass slides were transfected with YFP-mVP40, CFP-YAP1-WT, and/or CFP-YAP1 WW mutant plasmids for 20 hours. Cells were fixed with 4% paraformaldehyde (Affymetrix) and permeabilized with 0.1% TritonX-100, and then mounted with Prolong gold anti-fade mountant with DAPI (Thermofisher scientific-p36935). Images were obtained on a Leica SP5 inverted confocal microscope with a 100x (NA 1.46) objective lens. The confocal images were subsequently deconvolved with Huygens Essential deconvolution software. CFP-YAP1-WT and CFP-YAP1 WW mutant plasmids were kindly provided by K-L Guan.

### TIRF microscopy

HEK293T control (shCtrl) and Amot knockdown (shAmot) cells were plated onto coverglass bottom 35mm dishes (MatTek) and 24h later were co-transfected with plasmids expressing pCMV-LifeAct-RFP (ibidi) and YFP-mVP40. Just prior to imaging, the cells were stained with Deep Red CellMask plasma membrane stain (Invitrogen). Dishes were placed onto the stage of a GE DeltaVision OMX SR microscope and maintained at 37⁰C and 5% CO_2_ in a stage-top incubator. Approximately 20 cells per group were randomly chosen and imaged in SIM-TIRF mode. SIM super-resolution images were reconstructed from the acquired TIRF images.

### VP40 VLP budding assay

HEK293T, YAP KO, shCtrl, or shAmot cells in collagen-coated six-well plates were transfected with the indicated plasmids (total plasmid DNA was equivalent in all samples) using Lipofectamine reagent (Invitrogen) and the protocol of the supplier. At 6 hours post-transfection, OPTI-MEM was added for 24 hours. Culture media was centrifuged at 351 x g for 10 minutes to remove cellular debris, layered onto a 20% sucrose cushion in STE buffer and centrifuged at 220,000 x g for 2 hours at 4°C. The VP40 VLP pellet was suspended in STE buffer at 4°C overnight. The indicated proteins were analyzed by SDS-PAGE and Western-blotting, and proteins were quantified using NIH Image-J software.

### Protein-peptide docking

Protein-peptide docking analysis was performed using Glide module in Schrödinger. The protein structure of YAP WW1 domain (PDB ID: 2LTW) was downloaded from PDB [96] and was prepared with the Protein Preparation Wizard tool in Schrodinger. The peptides were built using Maestro and generated multiple conformers using the MacroModel sampling method. The receptor grid for peptide docking purposes was prepared with default settings using the peptide ligand to define the grid center. The peptide conformers were docked using Glide SP-PEP protocol [97].

### Filovirus stocks

All experiments with replication competent MARV were performed in the biosafety level 4 (BSL-4) laboratory at the Texas Biomedical Research Institute (San Antonio, TX). MARV strain Musoke (NCBI accession number NC_001608) was obtained from the virus repository at the Texas Biomedical Research Institute, San Antonio, TX [78].

### Filovirus spread

shCtrl and shAmot cells were grown in 96-well plates and incubated with MARV at MOI of 0.01 or 0.05. Three separate plates were challenged in triplicate for each MOI. After 1 h, inoculum was removed, cells washed with medium, and new medium was added to wells. Cells were fixed 24h, 48h, or 72h after virus challenge and then stained with Hoechst 33342. MARV-infected cells were also stained with anti-VLP antibody. Cells were photographed using a Nikon Ti-Eclipse microscope running high content analysis software (Nikon, Tokyo, Japan). The numbers of cell nuclei and infected cells were counted using Cell Profiler software (Broad Institute, Boston, MA).

### Filovirus release

To assess release of replication-competent MARV from shCtrl or shAmot cells, the supernatants of infected cells were harvested and overlaid onto monolayers of Vero cells (American Type Culture Collection; ATCC, Manassas, VA) grown in 96-well plates. After 24 h, cells were fixed, stained, and analyzed as above.

### Statistical analysis

Absolute intensities for each immunoblot lane were normalized to the intensity of the first lane of the blot and averaged across at least three replicates (collected from 3 or more separate transfections). Mean normalized intensity ±SD is shown. Due to observed inequality of variance between the groups (determined by F-test p-value <0.05), Welch’s t-test was used to determine statistical significance. For live virus data, mean percentage ±SD is shown from 3 independent experiments. Viral spreading was compared using the student’s t-test. All p values were designated using the following symbolic representation: ns = p > 0.05, * = p ≤ 0.5, ** = p ≤ 0.01, *** = p ≤ 0.001, **** = p ≤0.0001.

## Supporting information

S1 Table Host WW-Domains Interacting with the mVP40 PPxY Peptide. WW-domains from the listed host proteins were shown to interact with the PPxY motif present within mVP40.
(PDF)
