# Supplementary material for: Modular mimicry and engagement of the Hippo pathway by Marburg virus VP40: Implications for filovirus biology and budding
Source: PLoS Pathog. 2020 Jan 6;16(1):e1008231. doi: 10.1371/journal.ppat.1008231 (PMC6977764; doi:10.1371/journal.ppat.1008231)
Supplement: S1 Table — WW-domains from the listed host proteins were shown to interact with the PPxY motif present within mVP40. (PDF) [file ppat.1008231.s001.pdf]

**S1 Table: Host WW-Domains interacting with the mVP40 PPxY-peptide**
RSP5-1 Yeast WW
RSP5-2 Yeast WW*
Nedd4L-1 WW
Nedd4L-2 WW
Nedd4-2 WW
WWP1-1 WW
WWP1-2 WW
WWP1-4 WW
WWP2-1 WW
WWP2-2 WW
WWP2-3 WW
ITCH-4 WW
SMURF2-3 WW
YAP1-1 WW
YAP WW (XAV-clone 10)
WWOX-1 WW
MAGI2-1 WW
MAGI3-1 WW*
WWTR1 (TAZ) WW
BAG3 WW*
ARHGAP27-3 WW

*signal strength was strongest for these WW-domains

Proteins

RSP5 – yeast homologue of human Nedd4 E3 HECT ubiquitin ligase
Nedd4 – Neural precursor cell expressed developmentally downregulated gene 4 E3 Ub ligase
Nedd4L – Nedd4 Like E3 Ub ligase
WWP1 – WW domain containing E3 Ub ligase 1
WWP2 – WW domain containing E3 Ub ligase 2
ITCH – Itchy E3 Ub ligase (AIP4)
SMURF2 – SMAD Specific E3 Ub ligase 2
YAP1 – Yes-Associated Protein 1
WWOX - WW domain containing oxidoreductase
MAGI2 - Membrane-Associated Guanylate kinase Inverted 2
MAGI3 - Membrane-Associated Guanylate kinase Inverted 3
WWTR1 (TAZ) - WW domain containing Transcription Regulator 1
BAG3 – BCL2 Associated Athanogene 3
ARHGAP27 – Rho GTPase activating protein 27
